# Progress and challenges in predicting protein interfaces

**DOI:** 10.1093/bib/bbv027

**Published:** 2015-05-13

**Authors:** Reyhaneh Esmaielbeiki, Konrad Krawczyk, Bernhard Knapp, Jean-Christophe Nebel, Charlotte M. Deane

**Keywords:** protein–protein interaction, protein interface prediction, antibody antigen interaction

## Abstract

The majority of biological processes are mediated via protein–protein interactions. Determination of residues participating in such interactions improves our understanding of molecular mechanisms and facilitates the development of therapeutics. Experimental approaches to identifying interacting residues, such as mutagenesis, are costly and time-consuming and thus, computational methods for this purpose could streamline conventional pipelines. Here we review the field of computational protein interface prediction. We make a distinction between methods which address proteins in general and those targeted at antibodies, owing to the radically different binding mechanism of antibodies. We organize the multitude of currently available methods hierarchically based on required input and prediction principles to provide an overview of the field.

## Protein interfaces

Proteins interact with other proteins, DNA, RNA and small molecules to perform their cellular tasks. Knowledge of protein interfaces and the residues involved is vital to fully understand molecular mechanisms and to identify potential drug targets [1]. The most reliable methods to determine protein complexes and therefore protein interfaces are X-ray crystallography and mutagenesis. Unfortunately these techniques are expensive in time and resources. Therefore, over the past 25 years, there has been a rapid development of computational methods aiming to elucidate protein complexes, such as protein interaction prediction, protein–protein docking and protein interface prediction. These three types of methods all aim at slightly different problems, protein interaction prediction attempts to give a binary answer as to whether two proteins interact, docking aims to recreate the pairwise residue contacts between the two binding partners. The subject of this review is the middle ground between these two problems, protein interface prediction, where one wishes to identify a subset of residues on a protein, which might interact with the presumed binding partner.

Residues involved in these interfaces are normally defined by an intermolecular distance threshold (usually between 4.5 and 8Å [2] with the most common value being 5Å [3]) or a reduction of accessible surface area in a complex compared with the monomer [4] (Supplementary Figure S1 displays an example). Experiments have shown that the choice of interface definition has only a minor impact on a predictors’ performance [5]; the threshold values however are critical for selecting specific features of interfaces [6].

An interface residue predictor receives as input a protein or a pair of proteins. It then predicts a subset of residues on the proteins surface that are involved in intermolecular interactions. When comparing the true interacting residues with the prediction, it is standard to calculate the number of true positives (TP), false positives (FP), true negatives (TN) and false negatives (FN) (Supplementary Figure S2). These four values give rise to a variety of performance metrics (Table 1), which can be used to assess the quality of the predictor.

**Table 1.** Commonly used metrics to assess the quality of interface residue predictions

Metric	Formula
Specificity	$\frac{TN}{TN+FP}$
Sensitivity (also known as recall)	$\frac{TP}{TP+FN}$
Precision	$\frac{TP}{TP+FP}$
F1 (harmonic mean of precision and recall)	$\frac{2\times precision\times recall}{precision+recall}$
Accuracy	$\frac{TP+TN}{TP+TN+FP+FN}$
Matthews correlation coefficient (MCC)	$\frac{\left(TP\times TN\right)-(FP\times FN)}{\sqrt{\left(TP+FN\right)\times \left(TP+FP\right)\times \left(TN+FP\right)\times \left(TN+FN\right)}}$

A single interface prediction consists of a set of residues believed to constitute the binding site and those that do not. Out of those believed to be the binding site, if they are truly binding residues they are called TP, otherwise they are FP. Out of the residues identified as non-binding, if they do not constitute the interface, they are called TN and FN otherwise (see Figure S2). These four numbers are used to calculate a range of performance metrics presented in this table.

**Figure 1.**
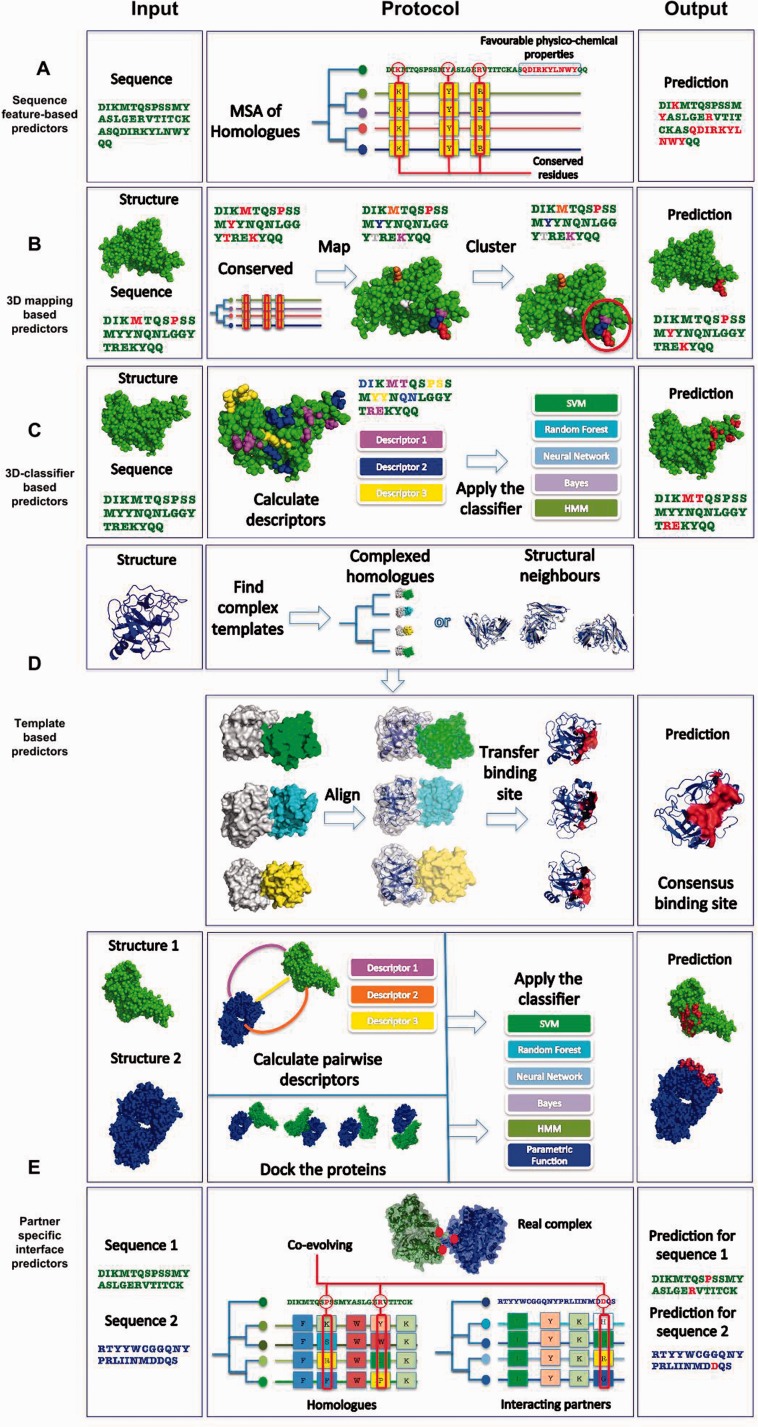
Classification of existing protein interface prediction methods. In the leftmost column we present the input required by a method. In the middle column, a simplified pipeline for the protocol is presented. In the rightmost, prediction column, the resulting binding site is shown in red. Most methods output a ranked list of possible binding sites. Here for simplicity, we show a single result for each method. (**A**) Sequence-feature-based predictors: These methods receive a protein sequence. Sequential features of the input are compared with features thought to contribute to a residue being part of an interface, such as conservation scores and physico-chemical properties. (**B**) 3D mapping-based predictors: These methods receive a protein structure and its sequence as input. Evolutionary conservation is coupled with 3D surface and sequence information. Conserved residues can be grouped according to their surface proximity to form contiguous interface patches. (**C**) 3D-classifier-based predictors: The input for these methods is a protein structure and its sequence. Distinct sets of attributes (physico-chemical, evolution, 3D structural features, etc.) are used as an input to a learning method such as a SVM or Random Forest. (**D**) Template-based predictors: These methods receive a protein structure (and thus its sequence) as input. Complex templates are then identified, which can be homologues or structural neighbours (these are shown in white, whereas their binding partners are in green, cyan and yellow). Templates of the input protein are aligned to the query protein. The most commonly aligned contact sites are returned as a prediction. (**E**) Partner-specific interface predictors: These methods receive the structures/sequences of two proteins that are assumed to interact. The three groups of methods are shown for this category. Partner-specific descriptors can be calculated to predict interfaces. In some cases docking is used to sample possible orientations to identify a consensus binding site. Partner-specific descriptors and docking poses are used as input for parametric functions and classifiers to obtain the final result. In the co-evolution-based strategy, a MSA of interacting homologues is created and sites that appear to mutate in concert (co-evolve) are assumed to constitute the binding site. A colour version of this figure is available at BIB online: http://bib.oxfordjournals.org.

The field of protein–protein interface prediction has diversified into many different approaches (Figure 1) [7]. Methods might use intrinsic features of the sequence or the structure, evolutionary relationships or use an existing complex as a reference template. Predictors make use of many distinct quality measures, different training and testing data sets, thus a fair comparison between them is hard [5]. In this review we attempt to provide a classification for the majority of existing methods in order to get a clear overview of the field. Based on this, we offer suggestions as to how the field could progress, focusing on improved predictions and unified evaluation metrics.

### Protein interface predictors

Computational methods for identifying interface residues can be broadly divided into two non-exclusive categories based on their use of protein information: (1) intrinsic-based approaches based on specific features of protein sequences and/or structures and (2) template-based approaches that exploit the conservation found between structurally similar proteins. A simplified overview of all methods is given in Figure 1, and detailed descriptions are provided in the subsequent sections along with a summary in Table 2.

**Table 2.** Protein interface predictors and their performance

		Input			Main knowledge source(properties)				Intrinsic-based			Template-based		Output		Performance								
Method	Predictor	Sequence	Structure	Both	Sequence	Structure	Both	Additional	Evolution Info.	Intrinsic features	Both	Homologous Structure	Structural Neighbour	Residue-based	Patch-based	Data set*	Recall %	Precision %	Specificity %	Accuracy %	MCC	F1 %	AUC	Numbers taken from*
A	[60]	x			x						x			x		[10]	45.55	86.98	97.41	83.12	0.55	59.79	–	
	[181]	x			x						x			x			57.9	–	65	62.5	0.22	52	–	
	[35]	x					x				x			x		[45]	83	–	78	–	0.76	–	–	
	[23]	x			x			+			x			x			47	22.2	69	66.4	0.13	25.6		
	[10]	x			x						x			x			42.84	81.96	–	–	–	56.25	–	
	[12]	x			x						x			x			70	37.7	–	–	–	49	–	[10]
	[22]	x			x			+			x			x		[23]	36.6	18.9	76.1	71.9	0.09	23.2	–	[23]
	[15]	x			x						x			x		[64]	69	–	65	–	0.28	67	–	[66]
	[16]	x			x						x			x			58.8	26.3	–	–	–	36.3		[10]
	[4]	x			x					x				x		[182]	39	–	58	72	–	–	–	
	[9]	x			x					x				x			50	62	–	–	–	10	–	[10]
B	[30]			x			x		x						x	[13]	39.8	–	86.9	72.6	–	–	–	
	[13] [183]			x	x				x						x	[13]	34.2	–	85.1	68.5	–	–	–	[30]
C	[68]			x			x				x			x		[71]	63.6	–	84.3	–	0.37	–	–	
	[65]			x			x				x			x		[64]	72.7	–	61	75.2	0.47	66.3	0.82	
	[71]			x			x				x			x		[184]	–	–	–	–	0.17	–	0.69	
	[54]			x			x				x			x			99.08	99.91	–	80.32	1.29	99.48	–	
	[57]			x			x				x				x	[45]	45.8	69.6	–	79.8	–	–	–	
	[58]			x			x				x				x		78.99	65.3	54.66	67.29	0.34	–	–	
	[66]			x			x				x			x	x	[64]	68	–	73	71	0.43	71	–	
	[55]			x			x				x			x		[50]	74.7	63.4	–	–	0.58	–	0.9	
	[39]			x			x				x			x		[185]	–	–	–	70	–	–	–	
	[49]			x			x				x			x		[64]	77	–	63	–	0.35	69	–	[66]
	[26]			x			x				x			x		[58]	78.27	63.44	51.28	65.3	0.30	–	–	[58]
	[64]			x			x				x			x			59	–	54	69	0.33	56	–	[66]
	[48]			x			x				x			x			60.7	–	41.9	–	0.20	–	–	
	[63]			x			x				x				x	[45]	–	–	–	–	–	–	–	
	[38]			x			x				x			x		CAPRI	41.7	40.3	–	–	–	–	–	
	[47]			x			x				x			x		[186]	46.2	42.2	–	83.2	0.30	44.1	–	
	[67]			x			x				x			x			37.7	57.8	–	75.1	0.31	45.7	–	
	[41]			x			x				x			x		CAPRI	30.1	30.4	–	76.9	0.16	30.2	0.60	[101]
	[70]			x			x				x			x		[64]	36	–	93	–	0.33	52	–	[66]
	[50]			x			x				x			x			60.3	63.7	–	74.2	0.42	–	–	
	[62]			x			x				x				x	–	–	–	–	–	–	–	–	
	[45]			x			x				x				x	–	–	–	–	–	–	–	–	
	[46]			x			x				x			x		[187]	67	22	–	67	–	–	–	
	[188]			x			x				x			x		CAPRI	34.5	37.4	–	79.5	0.23	35.9	0.71	[101]
	[34]			x			x				x			x			42.8	57.8	–	73.3	–	–	–	
	[61]			x			x				x				x	CAPRI	27.3	28.7	–	76.6	0.14	28	0.62	[101]
	[189]			x			x				x			x		[52]	–	–	–	76	0.5	–	–	
	[52]			x			x				x			x			–	–	–	72	0.43	–	–	[189]
	[51]			x			x				x			x		[48]	27.7	–	44.2	–	0.15	–	–	[48]
D	[72]															[186]	–	25	–	45	–	–	–	
	[74]															[186]	–	50.5	–	49.5	–	–	–	
																CAPRI	24	38.9	–	81.1	0.20	29.7	0.71	[101]
E	[90]			x			x					x		x		[184]	56.1	52.6	–	85.4	0.45	52.5	–	
	[88]			x			x					x		x		[190]	43	72.7	–	–	–	–	–	
	[27]		x			x						x		x			67.3	50	–	–	–	–	–	
F	[101]		x			x							x	x		CAPRI-bound	46.1	45.4	–	80.9	0.34	45.7	0.77	
																CAPRI-unbound	43.7	44	–	81.2	0.32	43.8	0.75	
	[99]		x			x							x	x		[190]	57.5	50.3	–	72.6	0.34	0.53	0.73	
																CAPRI-bound	53	43	–	72.1	0.29	0.47	0.71	
																CAPRI-unbound	53.6	43.3	–	73.2	0.30	0.48	0.72	
	[100]		x			x							x	x		[190]	45.7	43.60	–	–	–	–	–	
																CAPRI-bound	42.2	41.50	–	–	–	–	–	
																CAPRI-unbound	44.6	39.8	–	–	–	–	–	
	[97]		x		x					x			x	x	x	[98]	34	32	–	–	–	34	–	
	[98]		x		x					x			x	x	x		35.3	31.5	–	–	–	33.3	–	
G	[111]			x			x				x			x		[184]	–	–	–	–	–	–	0.47	
	[104]			x			x				x			x		[184]	–	–	–	–	–	–	0.87	
	[110]		x			x				x				x		[184]	62.2	40.4	–	–	–	–	–	
	[102]	x			x						x			x		[190]	–	–	–	–	–	–	0.72	
	[109]		x			x				x				x			72.7	39.3	–	–	–	51	–	
	[115]	x			x			+							x		–	–	–	–	–	–	–	
	[118]							x			x			x		[118] test	20	59	–	–	–	–	–	[118]
	[122]	x			x									x		[118] fitting	20	23	–	–	–	–	–	[118]
	[119]	x			x									x		[118] fitting	20	23	–	–	–	–	–	[118]
	[121]	x			x									x		[118] fitting	20	23	–	–	–	–	–	[118]
	[18]	x			x									x		[118] fitting	20	25	–	–	–	–	–	[118]
	[120]	x			x									x		[118] fitting	20	20	–	–	–	–	–	[118]

The predictors are grouped by their corresponding category from this manuscript, based on the input and methodology used. The numbers in the ‘Method’ column correspond to the heading numbering in the text (except from meta predictors). Performance measures, where available, were collected from the original publications. Where possible, the performance measures were taken from studies benchmarking several studies at once. Empty cells in columns with * correspond to the same study where its reference number is available in the predictor column in the same row. Cells with + refer to ‘predicted structural feature’. In the data set column, CAPRI refers to the targets used in the CAPRI challenge, which can be in the bound or unbound form. The 3D classifier group contains some methods, which are based on scoring function. Columns marked with x correspond to the features the predictor is using. Where data is not available - sign is used. In the Method column for ‘A' see section ‘Sequence Feature-based Predictors', for ‘B' see section ‘3D mapping-based Predictors', for ‘C' see section ‘3D-Classifier Predictors', for ‘D' see meta methods in section ‘Descriptors used by predictors', for ‘E' see section ‘Homologous Template-based Predictors', for ‘F' see section ‘Structural Neighbour-based Predictors' and for ‘G' see section ‘Partner-specific interface predictors'.

## Intrinsic-based predictors

### Sequence-based interface predictors

Sequence-based interface predictors use only the sequence features of the query proteins to detect interfaces and thus, can be applied to almost any protein. Early work exploited sequence features such as hydrophobicity distribution [8], composition/propensity to be an interface residue [9] and physico-chemical properties [4]. Predictors have also combined such features, using machine learning strategies such as support vector machine (SVM) [4, 10], neural-network [11] or random-forest [12]. Such approaches suffer from low specificity [4] and therefore later predictors proposed integration of evolutionary information to further improve prediction accuracy [4, 9].

#### Sequence feature-based predictors

The success of evolutionary information in predicting functional sites [13, 14] inspired many interface predictors to combine evolutionary information with other sequence features [15, 16]. Interface residues are more conserved than the rest of the protein surface [17, 18] and these conserved positions are identified from multiple sequence alignments (MSAs) [5, 18, 19] often with phylogenetic trees assisting the procedure [19–21] (Figure 1A).

The first predictor [16] that combined evolutionary information along with residue composition achieved an accuracy of 64%. This was a 6% increase over the previous sequence-based study [9]. Since then, several methods [12, 15] have experimented with a wide range of sequence-derived features combined with evolutionary information. However, the most recent method in this category [10] showed that using hydrophobicity alone combined with evolutionary information can achieve results similar to methods that use a far larger number of features [12].

In addition to evolutionary information, some sequence-based methods [22, 23] take advantage of predicted structural information (i.e. surface accessibility and secondary structure). Use of predicted structural information in ISIS [22] and PSIVER [23] increased the sensitivity of their predictions, for example, ISIS increased its sensitivity to 20% from a baseline of 0.5% [9]. These results demonstrate that inclusion of predicted structural information can increase the accuracy of interface prediction.

It appears that current sequence-based methods have reached their limit because further combination of available features does not improve accuracy. Therefore, alternative approaches and sources of information should be investigated. In particular, use of structural data has been shown to improve the performance of sequence-based interface predictors.

### Structure-based predictors

Structural features are important discriminative attributes for protein interface prediction. These features are associated with the atomic coordinate of the proteins, such as secondary structure [24, 25], solvent-accessible surface area [26, 27], geometric shape of the protein surface [26] and crystallographic B-factor [24]. Historically, methods using structural information were limited by the paucity of available 3D structures. However, in recent years the number of solved structures has been gradually increasing, enabling the development of 3D-based interface predictors. In these predictors, the query 3D structure is either used to identify interface residues in close proximity to each other (see the ‘3D mapping-based predictors’ section) and/or as structural features for detection of interface residues (see the ‘3D-classifier predictors’ section).

#### 3D mapping-based predictors

Conserved residues are an important source of information for interface predictors [28]. If the structure of the query protein is available, one can map the predicted/conserved residues directly onto the structure, identifying clusters of neighbouring residues [13, 28, 29]. This naïve use of structural information improves on sequence-only methods. In addition, including other physico-chemical attributes at the mapping stage can further increase prediction performance [30] (Figure 1B).

#### 3D-classifier predictors

Instead of considering structural information only at the mapping stage, 3D-classifier predictors use 3D structural features (or their combination with sequence features) directly to detect interfaces (Figure 1C). They exploit the fact that the binding interface has different structural properties when compared with the rest of the protein. For instance, Chothia and Janin (1975) [31] discovered that hydrophobicity is a key element to stabilizing protein–protein interactions, which inspired many of the early predictors in this category [24, 32, 33].

To investigate the importance of 3D information for detecting interface residues, predictions based on sequence information alone were compared with predictions including structural data [26, 34]. Results found that using structural information significantly improves prediction accuracy. This is probably mainly owing to the elimination of non-surface residues, greatly reducing the search space [35].

Not one single structural property completely discriminates interface residues from others. Therefore predictors have based their predictions on combining multiple input properties of residues. Methods in this category differ from one another by features employed and the methodology used to combine them. They are broadly divided in two groups [36] (i) score-based and (ii) probabilistic-based predictors. Predictors in both groups are trained using a training set to predict interfaces [36].

##### Score-based predictors

Score-based predictors calculate an interaction likelihood score for each residue. All residues with a score above a certain cut-off are classified as contacts [36]. Scores can be calculated from a linear [37, 38] or non-linear combination of sequence and structure contributions [36]. Features used include accessible surface area [39], Position Specific Scoring Matrix (PSSM), interface propensity and surface conservation [40], side chain energy scores [41, 42] or desolvation energy [43, 44]. The drawback of constructing such empirical functions is that they rely on specific knowledge of the physical system, which is often error-prone and not suitable for amendments and extensions [36]. This issue is tackled by non-linear combinations of features using machine learning techniques such as SVM [45–48], ensemble methodology [49, 50], Neural Networks [51–54] or Random Forests (RF) [55–59]. As the number of positive samples (interacting residues) is smaller than the negative samples, the training set for machine learning classifiers of interface and non-interface are imbalanced [59]. To deal with this problem, predictors have proposed strategies for splitting the training data into balanced subsets [10] and detecting outliers [60].

##### Probabilistic-based predictors

An alternative approach to using linear or non-linear combinations is to find the conditional probability $p\left(s\text{|}x_{1},\ldots ,x_{k}\right)$ of s being interface or non-interface, where $x_{1}$ to $x_{k}$ are the properties of the residue under study. Conditional probability can be generated from the training sets using Bayesian methods [61–63], Hidden Markov Model [64, 65] or Conditional Random Fields [66–68]. It has been argued that such probabilistic classifiers might offer an increased performance over the machine learning methods described above [62, 67].

##### Descriptors used by predictors

Machine learning techniques used by score-based and probabilistic-based predictors [59] provide a framework for evaluating the contributions of attributes to the predictive power. Previous studies have investigated which properties play an important role in the discrimination of interface and non-interface residues. The PSSM generated from PSI-BLAST [69] has been argued to be an important factor [47, 70] as well as solvent-accessible surface area, hydrophobicity, conservation and propensity [71]. It was also demonstrated that relative solvent accessibility has more predictive power than other features [50]. Recently it has been demonstrated that only four features, solvent-accessible surface area, hydrophobicity, conservation and propensity of the surface amino acids are sufficient to perform as well as the current state-of-the-art predictors [71]. To the best of our knowledge, the most recent benchmark of the predictive power of attributes was performed by RAD-T [59]. This study named relative solvent-excluded surface area and solvation energy as attributes with the most discriminative power. In the same study, it was established that among the different machine learning methods a random forest-based classifier performed the best. This best combination of attributes and the classifier currently forms the core of RAD-T.

Even though RAD-T performed a rigorous benchmark of the available methods and features to be employed, this predictor relies on one classifier, namely a variant of RF. It was argued that if predictors express a degree of orthogonality, they may be combined in a consensus-based classifier. Therefore, some methods have integrated individual interface predictors into one meta framework [72, 73]. For instance, meta-PPISP [74] combines the prediction scores of PINUP, Cons-PPISP and ProMate using linear regression analysis. One review study [36] confirmed the superiority of meta-PPISP over its constituent PINUP [41], Cons-PPISP [53] and ProMate [61] with accuracies of 50%, 48%, 38% and 36%, respectively.

While meta-predictors are an elegant way to improve the accuracy of individual constituents, significantly better performance is achieved only if the combination of features does not introduce redundancy [59, 75]. It appears that intrinsic-based predictors have reached saturation since further combination of existing features and classifiers has little impact on prediction performance [76]. Therefore, a complementary approach needs to be found in the form of new sources of experimental data or novel classifying methodology. This issue and an increasing number of structures in the Protein Data Bank (PDB) [77] have led to an emergence of an alternative trend in predictors, using existing complexes as templates for interface prediction.

## Template-based predictors

The growing number of available structural complexes assists accurate identification of interface templates. Studies have shown that interfaces are conserved among homologous complexes [78–81], inspiring the first category of template-based methods, which relies on homologous complexes. However such homologous structures are not always available. Therefore the second category of template-based predictors uses structurally, but not necessarily evolutionarily, similar complex templates.

### Homologous template-based predictors

These methods use known complexes where one of the interacting partners is homologous to the query protein. The interface via which the homologous protein interacts is assumed to be an indicator where the corresponding interface might be found on the query protein. This approach to interface prediction is possible, as it was demonstrated that homologous proteins tend to interact with their partners with a similar orientation [80] and the binding site localization within each family is often conserved regardless of the similarity of binding partner [78, 79, 81]. Physico-chemical properties of the interface residues have higher similarity in homologous proteins than non-homologous ones [82–86]. These observations suggest that integration of homologous structural information into interface predictors should improve performance. The current predictors in this category are HomPPI [35], IBIS [87–89] and T-PIP [90, 91].

HomPPI [35] builds an MSA of the query protein and its homologous complexes. Instead of looking at conservation at a residue level, HomPPI checks if the majority of the homologous residues at that position in the MSA are interface or non-interface. HomPPI implicitly takes advantage of binding site conservation of the homologous complexes. It performs better than 3D classifier methods such as ProMate [61], PIER [38], meta-PPISP [74], cons-PPISP [53] and PSIVER [23].

A combination of sequence and structure conservation scores was introduced in IBIS [87–89]. Initially, homologous complexes with at least 30% sequence similarity to the query protein are extracted. Then, these structures are superposed on to the query protein. Using this alignment, a structure-based-MSA is created, which allows the conserved interface residues to be identified. Comparison with HomPPI (62.8% precision and 50.4% recall) demonstrates the importance of using structure-based MSA (69.7% precision and 72.0% recall).

Recently, T-PIP [90, 91], which outperforms IBIS, was introduced (T-PIP with 52.6% precision and 56.1% recall and IBIS with 42.6% precision and 37.4% recall). Similar to IBIS it builds a structure-based MSA of homologues. The main novelty of T-PIP is that not only is the homology between the query protein and its homologues considered but also the diversity between the interacting partners of the homologues at each specific binding site.

In this category, the main attributes that appear to be contributing to the quality of predictions are the structure-based MSAs and the binding partner information. Although homologous template-based predictors improve the predictions over intrinsic-based methods, they are limited to those proteins where homologous complex structures exist. For instance, HomPPI has lower coverage than the 3D classifier methods and IBIS’s coverage is even lower. Although this issue has been partially addressed in T-PIP by lowering the threshold for selecting homologues, these predictors fail in cases where homologous complexes of the query protein are not available. This issue can be dealt with by using structural neighbours; complexes not necessarily evolutionarily related but with similar folds to the query protein.

### Structural neighbour-based predictors

Proteins sharing a similar fold with the query protein, even if not evolutionarily related, can offer similar predictive information to that of homologues. This was established by a study which found that functional relationship can be detected using remote structural neighbours [92]. Furthermore, proteins with similar folds but low sequence identity tend to interact with their partners using the same location [93, 94]. Such structural neighbours are exploited as templates for interface prediction to help overcome the low template coverage that can afflict homology-based methods (Figure 1D) [95–98].

Currently there are two main methods in this category, PredUS [99, 100] and PrISE [101]. PredUS is an earlier method, which identifies structural neighbours by finding structures with a globally similar fold to the query protein. PrISE, on the other hand, uses only the interface structure for template identification, which increases its prediction coverage. PrISE performance is similar to PredUS, as both methods achieve accuracy in the region of 81%. According to [101], PrISE performed better than methods that do not use template information.

In general, template-based methods show better recall scores, while intrinsic-based methods have better precision [90, 100, 101]. This suggests that intrinsic-based methods predict a smaller set of correct interface residues with higher confidence, which is especially important for mutagenesis studies. Also, T-PIP, a homology-based template method, has been shown to perform better (precision 52.6% and recall 56.1%) than the structural neighbour methods of PredUs (precision 47.3% and recall 58.2%) and PrISE (precision 38.5% and recall 48.9%). This improvement may be the positive impact of the consideration of interacting partners of the structural neighbours.

## Partner-specific interface predictors

The methods described above predict interfaces for one query protein, but proteins may display different interface patterns depending on their binding partner (e.g. antibodies [102]). Therefore, partner-specific predictors identify interacting residue pairs between two query proteins that are assumed to interact. One of the main challenges for these predictors is when unbound query protein structures are used. Therefore, performance of these methods decreases with the increase of conformational changes of the protein pairs on binding [102].

Partner-specific methods can be broadly divided into three groups, intrinsic-based methods, docking-based methods and co-evolution-based predictors. Intrinsic-based methods are similar in nature to the 3D classifier methods. The core difference is that the set of features that is being computed for training and testing is complemented by partner-specific features such as propensities and electrostatic complementarity [35, 102, 103]. The most recent method in this category is PAIRpred [104]. Application of these methods is seen in re-ranking docked decoys based on similarity to the predicted interface [90, 102, 105, 106].

Another type of approach uses protein–protein docking (Figure 1E) to generate potential interfaces (for a review on docking see [107, 108]). These methods generate docked poses of the two query proteins and detect interfaces based on contact energy and frequency scores [109]. The two main methods in this category are DoBi and RCF [110, 111]. DoBi (F-scores ∼ 0.55) outperformed the 3D classifiers such as MetaPPI, meta-PPISP, PPI-Pred, PINUP and ProMate (F-scores of 0.35, 0.43, 0.32, 0.43 and 0.21, respectively) [109]. While direct comparison between RCF and DoBi is not available, these results demonstrate the advantage of including partner information into the interface prediction. The main drawback is the requirement of the two protein structures. In addition, docking-based methods are slower, as generating docked poses is computationally expensive.

Co-evolution strategies have also been used to detect interfaces [18, 112]. The co-evolution principle suggests that mutations on one protein in a complex are often compensated for by correlated mutations within the same chain or on a binding partner. Such correlated mutations are assumed to maintain the stability of the protein or protein–protein complex [112]. By creating MSAs of the input proteins, one identifies the columns that appear to change in concert indicating spatial proximity. This paradigm has been used in protein structure prediction [113–116], scoring of docking decoys [117] as well as in protein–protein interface prediction [115, 118] (Figure 1E).

Early applications of co-evolution to protein interface prediction include OMES [119], MI [120] SCA [121], McBASC [18], ELSC [122] and the more recent i-Patch [118] and EVComplex [115]. The earlier methods generally suffer from low precision (20–25% precision at 20% recall) [118]. The more recent method, i-Patch, achieves higher precision (59%) for the same recall values, owing to the incorporation of structural information. The most recent method, EVComplex is capable of providing predictions from sequence alone, as it uses a structural model of the input. Its applicability was demonstrated by delivering interface predictions in accord with experimental data from a *de novo* model of ATP synthase complex. Co-evolution methods have over the past few years improved dramatically and this new approach has only just been tested on protein interface prediction. Since protein interaction data and sequence information is increasing exponentially, it is likely that this will further improve the quality and the applicability of co-evolution predictors in the future.

Predictors taking the binding partner into consideration [90] have shown promising avenues to better detection of binding sites. Therefore, predictors specialized to a specific type of protein such as antibodies may well yield better predictive power.

## Antibody–antigen complex modelling

**Figure 2.**
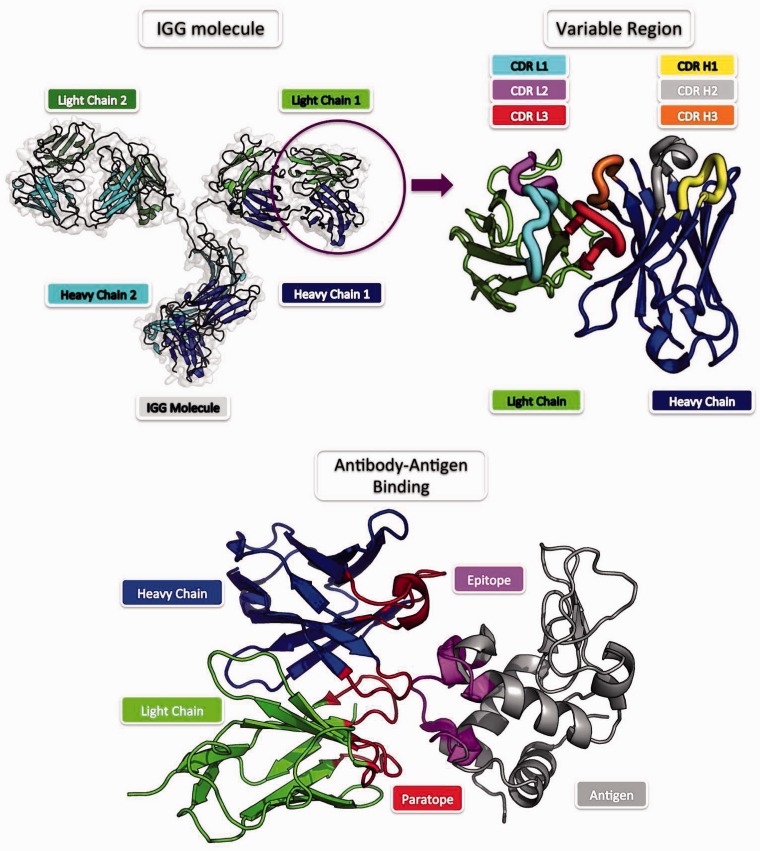
Antibody structure and binding. The most common form of an antibody is the IgG (upper left). IgG is composed of two pairs of heavy and light chains. The tip of an antibody that carries the binding site (symmetrical in an IgG) is the variable region (upper right). The variable region harbours the six CDR loops, which form the majority of the antigen recognition site, the paratope (lower). The CDR regions are distinct between different antibodies whereas the rest of the antibody remains largely unchanged. The paratope recognizes a specific epitope, the corresponding binding site on the antigen (lower). A colour version of this figure is available at BIB online: http://bib.oxfordjournals.org.

Antibodies are currently the most important class of biopharmaceuticals [123]. The success of antibodies as therapeutics depends on their intrinsic binding mechanism, which allows them to be adjusted toward almost any antigen target by mutations in a well-defined binding region (see Figure 2). The antibody–antigen binding mechanism is radically different to that of general proteins [124] and thus methods attempting antibody–antigen interaction prediction have developed into a separate domain [124–127]. Antibody–antigen interface predictors can be broadly classified into methods that predict the binding residues on either the antibody (paratope prediction) [128] or the antigen side (epitope prediction) [129].

### Paratope prediction

The antibody binding site is chiefly composed of six loops known as complementarity determining regions (CDRs). These CDRs have been described using a variety of definitions [127, 130–133], which suggest they contain between 40 and 50 residues. Examinations of antibody complexes show that there are on average 10–15 paratope residues, the majority of which are within the CDRs.

It was recently demonstrated that the residues contained within the boundaries of these CDRs contain only about 80% of the paratope [127]. On the basis of this finding a more robust definition of the antibody binding region was introduced and implemented—PARATOME [127]. Given a sequence or structure of an antibody, PARATOME aligns sequentially similar antibodies with solved complexes. The contacts from the aligned sequences are used in a consensus score to define the binding region for the query. This methodology maximizes the recall (∼94%) at the cost of precision (∼30%) because, just as the CDR definitions, it generates an annotation for the entire binding region neighbourhood rather than singling out possible contact residues.

In contrast to region-wide annotations given by CDR definitions and PARATOME, over the past 2 years there has been an increasing interest in developing methodologies that predict specific paratope residues. There are currently three methods which address this problem: proABC [128], Antibody i-Patch [124] and ISMBLab-PPI [134]. ProABC is a RF-based machine learning protocol, which requires only the sequence of the antibody on input. Antibody i-Patch is a statistical method, which relies on the structure of the antibody; however, it was demonstrated that it is robust to the use of homology models. The most recent method, ISMBLab-PPI, is a neural-network protocol. In contrast to proABC and Antibody i-Patch, its training set is not restrained to antibody–antigen complexes only. This might explain why it underperforms against proABC (comparison with Antibody i-Patch was not performed).

The field of paratope residue contact annotation appears to be greatly underdeveloped, mostly as a result of the assumption that knowing the CDRs is sufficient for antibody engineering through mutagenesis. The antibody binding region however contains on average 40–50 residues and thus complete mutagenesis of this entire region is currently not tractable while only around 18–19 residues are in contact with antigen [135]. For this reason, knowledge of particular paratope residues that might be important for binding would greatly reduce the search.

### Epitope prediction

Identifying regions on the antigen that are capable of binding an antibody is an important problem from the point of view of vaccine development and immunogenicity [136–138]. This is particularly difficult because epitope patches appear to be barely distinguishable from general protein surfaces [126, 134, 139]. There exist several experimental methods to identify epitope residues but all of them are costly in time and resources. For this reason, the field of computational B-cell epitope prediction has been developed intending to provide information on potentially immunogenic structures and sequences.

Computational epitope predictors can be divided into linear and conformational predictors. Linear epitope predictors aim to identify contiguous stretches in the antigen sequence, which constitute the epitope, while conformational ones focus on identifying patches of sequence on the antigen, which, when folded, constitute the linearly discontinuous epitope. Around 90% of all known epitopes are conformational [139]. Nevertheless, most of the methods developed over the past 20 years addressed the easier problem of linear epitope identification [129, 140]. Here we focus exclusively on conformational epitopes.

### Classes of conformational epitope prediction

Conformational B-cell epitope predictions can be classified into two types, those using antibody information and those that do not. The vast majority of them do not use any antibody information (e.g. CEP [141], DiscoTope [142, 143], ElliPro [144, 145], PEPITO [146], PEPOP [147], SEPPA [148, 149], EPITOPIA [150] and others [151, 152]). Consensus-based methods such as EPCES [153] or the meta-server EPSVR/EpMETA [154] are currently among the best-performing algorithms in this area [152].

### Data resources for epitope prediction

The main aim of methods that use no antibody information is to identify epitope-like sites on proteins as a means to improve vaccine design. Their mode of operation is similar in nature to that of general protein–protein interface prediction introduced in the earlier sections. In contrast to general protein predictors, epitope predictors use antibody-antigen-specific data from the PDB, AntigenDB [155], the Conformational Epitope Database [156], DIGIT [157], Immune Epitope Database [158–160], IMGT [161], Structural Antibody Database [162] and others [163]. The main issue is that virtually any part of a protein can be an epitope for some kind of a monoclonal antibody; thus including antibody information may be crucial [125, 164].

### Antibody-specific epitope prediction

The field of antibody-specific conformational B-cell epitope predictors is relatively underdeveloped—only six methods exist to address this problem [125, 164–168]. The earliest used only 26 antibody–antigen complexes (those available in 2007) to produce its predictions [165]. They combined the program FADE [169] for paratope–epitope complementarity with FastContact [170] for physicochemical descriptor calculations. On their small test set they achieved 18% sensitivity and 87% specificity.

Another method that attempted to obtain antibody-specific predictions relied on the coupling of ASEP and DiscoTope [166]. The ASEP potential was computed by counting residue–residue interface preferences from a non-redundant set of antibody–antigen complexes from the PDB. This potential was then used to constrain general epitope predictions made by DiscoTope, with respect to a single antibody.

Following their study of antibody–antigen complexes [167, 171], Zhang *et al.* developed a method that treats antibody–antigen interactions as a Hidden Markov Model. They used 80 antibody–antigen complexes to train their method, achieving 43% sensitivity and 71% specificity. The testing procedure was performed using leave-one-out validation, which, as the authors admit, given the redundancy of their data set might have led to over-fitting [167].

Recently a mixed computational-experimental method was proposed to predict antibody-specific epitopes [164]. An RF-based computational method assesses the propensity of possible antibody–antigen residue matches to be in contact. Their first protocol, ‘per-residue’, requiring sequence of the antibody and structure of an antigen outperforms EPSVR, which relies on the antigen structure. Their second protocol, ‘patch-per Ab’, requiring the structure of an antigen, performed even better. They demonstrated its application in combination with blocking experiments in making good predictions for the antibody D8 for VACV. Such combination of computational and experimental techniques holds a particular promise in being able to identify epitopes with a much higher throughput than crystallization.

The most recent general antibody-specific epitope predictor is EpiPred [125]. Its protocol requires the structure of an antibody (which can be a homology model) and the structure of the antigen. Antigenic epitopes are identified by performing simplified surface matching complemented by antibody-antigen-specific statistical scoring. This method (44% recall at 14% precision) outperforms the antibody-ignoring Discotope (23% recall at 14% precision), demonstrating the value of introducing antibody information into predictions.

There has not yet been a comprehensive study benchmarking the antibody-specific methods. Because antibody information improves the quality of predictions, we expect the field to investigate further antibody-specific predictions. One of the main challenges remains the lack of understanding of antibody specificity. A comprehensive study contrasting different epitopes on a single antigen (e.g. lysozyme) with respect to their binding antibodies could improve our understanding of the specificity of antibodies, providing ground for better epitope predictions.

## Conclusion

In this review we have discussed the myriad features and techniques used by protein interface predictors (summarized in Table 2). Although considerable effort has been expended to develop the field thus far, no method yet yields excellent results and objective comparison between approaches is difficult.

However, usage of 3D structural and evolutionary properties tends to improve results over predictions based on sequence alone. It appears that feature-based methods have reached saturation, and the inclusion of more properties does not improve predictive performance. A possible solution to this problem would be to diversify the predictions into specific protein types, such as antibodies, kinases and GPCRs. Such predictions would exploit the intrinsic features of these particular protein complexes, a property that is lost if all the proteins are considered together [172].

With the increasing availability of structural templates [173, 174], a new trend in protein interface prediction methodology uses structural homologues or structural neighbours for template-based predictions. Although, in many cases, the binding partner of the template is disregarded, taking it into account could contribute to better predictive power in a similar way as knowledge of the antibody contributes to epitope prediction.

Furthermore the increasing amount of complex structural data available has made it possible to perform large-scale protein–protein interaction predictions [175–178]. As such proteome-scale approaches are one novel way to address the protein interface prediction problem.

Benchmarking of protein interface prediction methods has so far not been systematic. Because predictors are assessed on different data sets by distinct metrics, it is currently difficult to fairly evaluate the multitude of methods and identify clear areas for improvement. This would be facilitated if protein interface predictors consistently formed a subcategory in the Critical Assessment of Prediction of Interactions (CAPRI) challenge [3, 179, 180, 191] or developed their own assessment scheme. Thus, introducing unified training and test data sets as well as blind benchmarking is essential for the further development of the field.

**Key Points**

- There is a plethora of available protein interface predictors and the field in its current state appears to be saturated. This calls for new methodologies or sources of information to be exploited. Recent methods use existing complexes as templates or use co-evolution to inform predictions.
- One avenue of recent interest is the specialization of methods with respect to a single protein type, e.g. antibodies, which could improve predictions and make benchmarking more transparent.
- There is an urgent need to benchmark the available methods in a consistent manner. Available protocols rarely perform comprehensive comparisons. Therefore it is impossible to precisely identify areas where improvement is necessary. Consistent participation of available predictors in the CAPRI challenge or development of a protein interface predictor-specific assessment scheme would address this issue.

## Supplementary data

Supplementary data are available online at http://bib.oxfordjournals.org/.

## Funding

2020 Science Programme (UK Engineering and Physical Sciences Research Council (EPSRC) Cross-Discipline Interface Programme, EP/I017909/1).

## Supplementary Material

Supplementary Data
