# Supplementary material for: Progress and challenges in predicting protein interfaces
Source: Brief Bioinform. 2015 May 13;17(1):117–31. doi: 10.1093/bib/bbv027 (PMC4719070; doi:10.1093/bib/bbv027)
Supplement: Supplementary Data [file supp_17_1_117__index.html]

Progress and challenges in predicting protein interfaces — Progress and challenges in predicting protein interfaces — Supplementary Data 

# Progress and challenges in predicting protein interfaces

## Supplementary Data

files

**Files in this Data Supplement:**

- Supplementary Data - docx file
- Supplementary Data - docx file
