# Supplementary material for: Progress and challenges in predicting protein interfaces
Source: Brief Bioinform. 2015 May 13;17(1):117–31. doi: 10.1093/bib/bbv027 (PMC4719070; doi:10.1093/bib/bbv027)
Supplement: Supplementary Data [file supp_bbv027_New_Microsoft_Office_Word_Document.docx]

**Additional files:**

**Figure S1: Example of a pair of interacting proteins (PDB code 1AVX)**. Two definitions of the binding interface are presented: distance-based (left) and accessible-surface-area-based (right). Most often the two definitions are used in conjunction. Below the structure representation, one can see the color-coded regions in the sequence, defining the discontinuous binding interfaces.

**Figure S2**. **Example of quantification of interface prediction**. ‘Solution’ refers to the native-contacts with respect to a solved complex at a certain distance and accessible surface area cutoff. ‘Prediction’ is an annotation with putative interacting residues according to an interface predictor. Contrasting the solution with the prediction gives rise to four values, true positives (TP) (predictor correctly identified contact residue), false positives (FP) (predictor annotated non-interacting residue as interacting), true negatives (TN) (predictor correctly annotated non-interacting residue) and false negatives (FN) (predictor annotated interacting residue as non-interacting).
